# The contribution of circadian clock to the biological processes

**DOI:** 10.3389/fmolb.2024.1387576

**Published:** 2024-06-06

**Authors:** Beibei Luo, Jiangyuan Song, Jiaqi Zhang, Jun Han, Xin Zhou, Lili Chen

**Affiliations:** ^1^ Department of Stomatology, Union Hospital, Tongji Medical College, Huazhong University of Science and Technology, Wuhan, China; ^2^ School of Stomatology, Tongji Medical College, Huazhong University of Science and Technology, Wuhan, China; ^3^ Hubei Province Key Laboratory of Oral and Maxillofacial Development and Regeneration, Wuhan, China

**Keywords:** circadian clock, circadian rhythm, growth and development, metabolism, biological processes

## Abstract

All organisms have various circadian, behavioral, and physiological 24-h periodic rhythms, which are controlled by the circadian clock. The circadian clock controls various behavioral and physiological rhythms. In mammals, the primary circadian clock is present in the suprachiasmatic nucleus of the hypothalamus. The rhythm of the circadian clock is controlled by the interaction between negative and positive feedback loops, consisting of crucial clock regulators (including Bmal1 and Clock), three cycles (mPer1, mPer2, and mPer3), and two cryptochromes (Cry1 and Cry2). The development of early mammalian embryos is an ordered and complex biological process that includes stages from fertilized eggs to blastocysts and undergoes important morphological changes, such as blastocyst formation, cell multiplication, and compaction. The circadian clock affects the onset and timing of embryonic development. The circadian clock affects many biological processes, including eating time, immune function, sleep, energy metabolism, and endocrinology, therefore, it is also crucial for overall health, growth and development after birth. This review summarized the effects of the circadian clock in the body’s physiological activities. A new strategy is proposed for the prevention of malformations or diseases by regulating the circadian clock or changing circadian rhythms.

## Introduction

The circadian clock is organized in a hierarchical manner and is distributed within organs, cells, and tissues throughout the body. The central clock, known as the suprachiasmatic nucleus (SCN), is at the top of this hierarchy (Moreno et al., 2019). Several prokaryotes and most eukaryotes, whether bacteria or humans, have circadian clocks that manifest in the daily rhythms of physiology, behavior, and biochemistry (Rida et al., 2004). The mammalian circadian clock consists of a set of transcriptional circuits that oscillate in a 24-h cycle to maintain the rhythm of metabolism, sleep-wake cycle, and cell proliferation (Miller et al., 2007). The circadian clock receives daily signals from external environmental sources, including darkness and daylight, to drive circadian rhythms. Ultimately, circadian rhythms control the normal physiological, behavioral, and biochemical transmission of the organism (Maiese, 2017). In mammals, the length of a cycle of circadian rhythms is determined by the feedback loop regulation of circadian genes *per* (*1* and *2*), *clock*, *cry* (*1* and *2*), and *bmal1* transcription in the middle of the circadian rhythm within the SCN. The length of this time is the transcription of *cry 1 and 2* and *per 1 and 2*, starting with *clock* and *bmal1* (Amano et al., 2020). The circadian clock cycle in mammals (including humans) is approximately 24 h. Therefore, circadian clocks require external input (time hints) to move phases to synchronize (or implicate) the environment (for example, light/dark cycles) (Moore and Lenn, 1972). The circadian clock receives optical and non-optical signals (Morris et al., 2012). Changes in eating habits, such as eating late at night, can result in inconsistencies between peripheral and central clocks. The optimal function relies on proper alignment between the central circadian clock (the SCN), chiaroscuro period, behavioral outputs (for example, activity, sleep, and diet), and the peripheral clock (Hastings et al., 2003). The development of early mammalian embryos is an ordered and complex biological process that includes stages from fertilized eggs to blastocysts and important morphological changes, such as blastocyst formation, cell multiplication, and compaction (Yong et al., 2021). Circadian rhythms extensively control physiological and pathological processes, including various human diseases and embryonic development in mammals (Jensen and Cao, 2013). The development and growth of the body are complicated processes that are affected by a number of factors, such as genetic background, the endocrine system, and environmental conditions (Cirillo et al., 2020). Studies have shown that the circadian clock affects many biological processes, including eating time, immune function, sleep, energy metabolism, and endocrinology (Luo et al., 2021). Healthy sleep is essential for overall health, development, and growth (Blaty and DelRosso, 2022). Our review explores the influence of the circadian clock to the embryonic development and body growth and development. It has also been shown that 2%–10% of mammalian genes are clock-controlled, and that they are mostly related to organ function and exhibit tissue-specific expression (Chu et al., 2011). Studies have shown that the circadian clock function is important for the cell cycle, *in vivo* tumor suppression, and DNA damage responses (Fu and Lee, 2003). Disorders of circadian rhythms can lead to serious health problems such as metabolic syndrome, organ fibrosis, aging, cancer, and osteopenia (Egstrand et al., 2020) These studies show that the circadian clock can not only regulate the growth and development of embryos and organs, but also has important implications for body health and disease development. Therefore, our review discusses the influence of circadian clock on the life process of the body, and provides new strategies and new ideas for the prevention and treatment of malformations and diseases.

## Effect of the circadian clock on embryonic development

The circadian clock affects the onset and timing of growth and development (Dunlap and Loros, 2006; Dunlap et al., 2007). It has been suggested that the cell cycle itself may be a degenerate circadian clock (Hunt and Sassone-Corsi, 2007). Light affects the circadian rhythms of all mammals (Dauchy et al., 2018). Other environmental factors such as temperature fluctuations, maternal nutrition, and stressors also influence circadian rhythm (Risely et al., 2021; Thompson et al., 2021; Tchekalarova et al., 2022). Important findings by Weiss et al. suggested that circadian rhythms of the eye may be involved in the regulation of eye growth (Chakraborty et al., 2018). The photoperiod, a seasonal and daily oscillating rhythm, can delay or inhibit sexual maturation and alter the ovarian developmental cycle (Dickey and Swanson, 1998; Rodríguez et al., 2005). Several previous studies have shown that both reducing and increasing light affect follicle development compared to a normal photoperiod (Shahed and Young, 2013; Murphy et al., 2015). Other studies have shown that associated signaling pathways and the photoperiod affect the secretion of sex hormones and development of the reproductive system (Vasal and Sundararaj, 1976). Numerous studies have reported that long-term (more than 6 weeks) exposure to light can affect ovarian development in rats, sheep, mice, cattle, and other mammals (Platt et al., 1983; Amaral et al., 2014). Based on the reflection of light by circadian rhythms, a prolonged artificial photoperiod can affect ovarian development (Chakraborty et al., 2018). In breast tissue, the expression pattern of the core clock gene changes in the female reproductive state and appears to be related to changes in the glandular development stage (Metz et al., 2006; Casey et al., 2014). Sleep disorders are often associated with children with neuropsychiatric disorders, and persistent sleep disorders can lead to impaired neuronal damage and brain development (Alfano and Gamble, 2009; Baddam et al., 2018). The interaction of clock genes and synapses may be involved in the pathogenesis of neuropsychiatric disorders, although sleep is considered to be necessary for brain maturation and synaptic development (Landgraf et al., 2014). Circadian rhythm disturbances affect brain stem development. Typically, Autism spectrum disorder first appears in infancy. Autism spectrum disorder is now considered a neurodevelopmental disorder. spatiotemporal disturbances of brainstem development may be the primary cause of Autism spectrum disorder that spread to the cerebral cortex (Takumi et al., 2020). Circadian rhythm disturbances are considered to be a common factor in developmental models of neuropsychiatric disorders (Marco et al., 2016). Sleep and circadian rhythms are constantly changing over the course of development, the dynamic relationship between sleep and circadian rhythms is necessary to understand sleep problems associated with ASD throughout development (Glickman, 2010).

**FIGURE 1:**
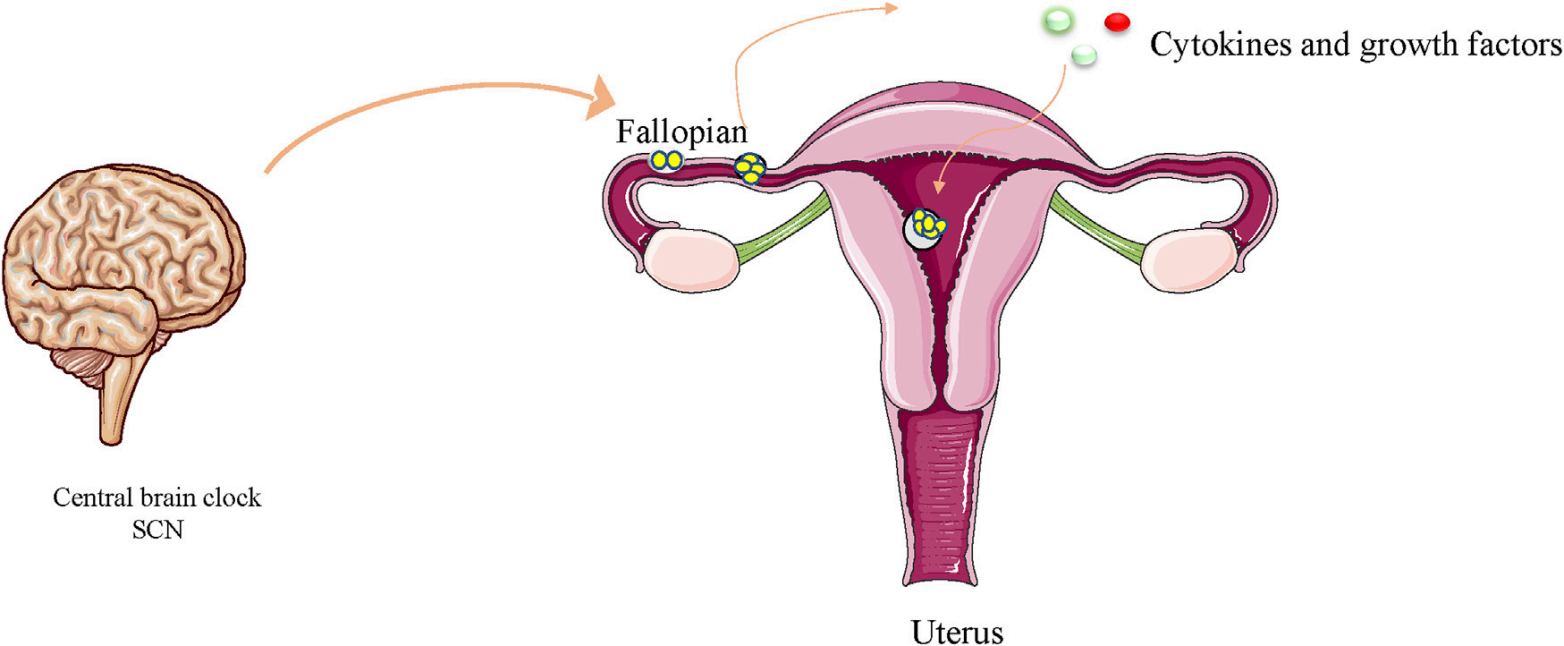
Circadian oscillators have been reported to guide early embryo division. Cytokines and growth factors secreted and expressed by the embryo itself may be influenced by circadian transcription factors, thereby exerting autocrine control over embryonic development and paracrine stimulation of endometrial tolerance.

The circadian clock transcription system has been reported to regulate cell proliferation intricately at the epigenetic level (Miller et al., 2007). The clock genes CLOCK (NPAS2) and BMAL1 may be the most important regulators of circadian rhythm. After translation and activation, they trigger the transcription of other CCGs. The dimerization of CLOCK and BMAL1 is achieved by helix-loop-helix motif on the per-arnt-sim domain of the CLOCK protein. Further epigenetic alterations, sumoylation, and phosphorylation of BMAL1 (Kondratov et al., 2003; Cardone et al., 2005) enhance the function of both proteins. After dimerization, they enter the nucleus to trigger the expression of PER and CRY genes (Gekakis et al., 1998). DNA methylation plays an important role in the regulation of PER2 gene and dysregulation can lead to metabolic disorders (Milagro et al., 2012). PER is thought to be phosphorylated by casein kinase I (CKI), while CRY is phosphorylated by adenosine monophosphate activated protein kinase (AMPK) (Lamia et al., 2009). CKI mutations lead to shorter daily cycles due to the accumulation of undegraded PER proteins, whose high levels eventually lead to an acceleration of the circadian rhythm, whose high levels eventually lead to an acceleration of the circadian rhythm (Lowrey et al., 2000). A gene-based approach has detected significant associations of CpG methylation pattern in PER2 gene with blood glucose and insulin resistance (Peng et al., 2019). Similar observations were made for the CLOCK and BMAL genes. Another result suggests that Bmal1 controls gene expression in inflammatory activation responses by regulating the epigenetic status of enhancers (Oishi et al., 2017). There is evidence that exposure to air pollution particles during pregnancy alters the methylation status of core circadian factors (CLOCK-BMAL1) and may be associated with circadian disruption (Nawrot et al., 2018). Nocturnin has been shown to be a clock-controlled deadenylase in mammals (Baggs and Green, 2003). Satoshi NISHIKAWA et al. found that early embryos and MⅡ oocytes of mice expressed nocturnin in circadian rhythms, and that accurate expression of nocturnin is imperative for the normal pre-implantation development of embryos (Nishikawa et al., 2013). Disrupting the circadian rhythm of the mother through exposure to chronic phase shifts in the photoperiod can have a lifelong effect on fetal metabolic homeostasis, leading to hyperinsulinemia in the offspring, increased obesity, and decreased insulin and glucose tolerance (Varcoe et al., 2013). Clock gene transcripts are components of maternal genetic transcripts and are often required for the first stage of embryonic development (Vatine et al., 2011). There is evidence that light can increase embryonic and post-hatch weights and pectoral muscle weight (Rozenboim et al., 2004). The nucleoplasmic shuttle of the clock in the E13–15 pancreas may be involved in the timing of pancreatic (Varcoe et al., 2013)rate of differentiation, which are regulated by circadian elements such as the clock and time (Li et al., 2007). Changes in light conditions (spectrum and intensity) affect the development of fish larvae and embryos in an underwater environment, and persistent red light or dark/light can lead to increased mortality and deformities (Sánchez-Vázquez et al., 2019). Tamara J. Varcoe et al. showed that pregnant female mice exposed to chronic phase shifts in the photoperiod profoundly alter the metabolism and expression of circadian rhythm genes in fetuses and mothers, potentially negatively affecting developing fetuses (Varcoe et al., 2013). Shift work of mothers can severely affect food consumption and the timing of activities, which can have a lasting impact on developing babies, making these offspring susceptible to metabolic disorders in adulthood (Varcoe et al., 2013). Tao et al. found that disturbances in circadian rhythms in pregnant mice also delayed enamel development in babies (Tao et al., 2016). Circadian oscillators have also been reported to guide early embryonic development. Cytokines and growth factors secreted and expressed by the embryo itself may be influenced by circadian transcription factors, thereby exerting autocrine control over embryonic development and paracrine stimulation of endometrial tolerance (Hardy and Spanos, 2002; Vlismas et al., 2016) (Figure 1). Maternal hormonal disturbances due to circadian rhythm changes or lack of sleep may disrupt fetal growth (Roman and Karlsson, 2013) (Table 1). *Per3* is a main components of the circadian clock system. Mariko Noda et al. showed that the haploid dysfunction of *Per3* may be associated with the onset of neuropsychiatric disorders, and *Per3* plays an important role in brain development. In the neocortex of mice, the circadian clock gene regulates the onset of brain development after birth. In the developing mouse cerebral cortex, when *Per3* was silenced, in addition to the abnormal position, the formation of dendritic protrusions and the projection of axons between the brain hemispheres were also canceled (Noda et al., 2019). As for the functional significance of circadian clock proteins in cortical development, oligophrenin-1, which is known to regulate dendritic spine morphology, interacts with Nr1d1 and is involved in neuronal function and structure during brain development (Valnegri et al., 2011). Lasse Dahl Jensen et al. recently found that circadian oscillations control angiogenesis in a developing zebrafish embryo model (Jensen and Cao, 2013). *Bmal1* and *Per2* have been reported to play roles in the regulation of angiogenesis in zebrafish development (Jensen et al., 2012). Jensen LD et al. exposed FLI1:EGFP zebrafish embryos to 12 h dark/12 h light (LD), persistent dark (DD), or persistent light (LL) environments. Exposure of zebrafish embryos to LL, in contrast to DD and LD, resulted in delayed angiogenesis of intersegmental vessels (ISV). DD exposure did not markedly alter ISV growth compared to that of LL. These results show that circadian rhythm disturbances may regulate angiogenesis in zebrafish embryos (Jensen et al., 2012). Studies have shown that the function of *Bmal1* is inhibited by its specific morpholine, resulting in significant inhibition of ISV growth in zebrafish embryos (Jensen and Cao, 2013; Dunlap et al., 2007). In *drosophila* (Allada et al., 1998) and mice (King et al., 1997), homozygous deletion of the clock gene is fata to embryos. Throughout the embryonic development process, the core circadian rhythm genes represented by *Clock* are expressed in harmony with tissue differentiation and can be used as a timing device through regulation of Notch- and Wnt-based components and cell cycle regulation (Li et al., 2007). Studies have reported that the *Clock* gene *Timeless* is involved in branching morphogenesis in mouse urethral buds (Xiao et al., 2003). Homozygote *Tim (−/−)* embryos die early in the womb, with a general lack of cell tissue and necrotic cell death (Gotter et al., 2000). Rigzin et al. used genome-wide methods to identify indirect and direct targets for the clock-controlled transcription factor ADV-1 and light response in *Brucella*. A large proportion of the ADV-1 target was found to be clock-controlled and rich in genes associated with development and cell growth (Dekhang et al., 2017). In peripheral tissue cells, the circadian clock regulates gene expression by regulating circadian rhythms, such as *Mdm2*, *C-myc*, *Cyclind1*, *P53*, and *Gadd45a*, controlling apoptosis and proliferation (Hunt and Sassone-Corsi, 2007). *Clock-△19* mice exhibited a point mutation that led to the downregulation of the Clock-Bmal1 target gene, which manifested as a decrease in the growth rate of young mice and an increase in neonatal mortality (Kennaway et al., 2004; Dolatshad et al., 2006). In contrast, melatonin promotes mitochondrial biogenesis by activating the SIRT1/PGC-1α pathway, compensating for energy deficiency and mitochondrial depletion, and promoting early embryonic development (Niu et al., 2020). The circadian clock and Smad2/3/4-mediated nodal signals regulate several pathological and physiological processes. Sha-Sha Bian et al. found that *Clock1a* coordinates the original blood supply and development of the mesoderm in zebrafish embryos by directly upregulating the Nodal-Smad3 signal (Bian et al., 2017). It has been reported that oscillations of circadian gene expression may serve as the master controllers of other potential timing oscillators, such as WNT and Notch signaling rings, as well as cell cycle regulation, and as the main regulator of the embryonic differentiation clock (Li et al., 2007). Studies have shown that interruptions in molecular clocks or changes in light conditions lead to changes in INR/TOR signals, which impact the growth and proliferation of neural stem cells in the brain of developing *Drosophila* (Dapergola et al., 2021).

**TABLE 1:** Effect of the circadian clock on embryonic development.

Mouse/Experiment/Mutation	Phenotype/mechanism	References
*Clock-△19* mice had a point mutation	downregulation of the *Clock-Bmal1* target gene led to increasing in neonatal mortality	Kennaway et al. (2004)
Melatonin	affects the formation of teeth, bones, adrenal glands and testicles	Kasahara et al. (2010)
Per3	plays an important role in brain development	Noda et al. (2019)
Sleep disorders	leads to impaired brain development and neuronal damage	Alfano and Gamble (2009)
Clock1a	coordinates the original blood supply and embryonic development of the mesoderm in zebrafish embryos by directly upregulating The Nodal-Smad3 signal	Bian et al. (2017)
Bmal1 and Per2	play a role in the regulation of angiogenesis in zebrafish development	Jensen et al. (2012)
Tim (−/−)	The embryo dies prematurely *in utero*	Gotter et al. (2000)
Local or integral deletion of fetal *Bmal1*	produces recognizable phenotypic abnormalities	Dan et al. (2020)
*Bmal1* tissue-specific mutations	circadian clock dysfunction itself may contribute to congenital defects	Dan et al. (2020)
Nr1d1	involved in neuronal function and structure during brain development through interaction with Oligophrenin-1	Valnegri et al. (2011)
Bmal1 defect	results in a significant inhibition of the growth of ISV in zebrafish embryos	Jensen and Cao (2013)
Clock	Throughout embryonic development, it can be used as a timing device by modulating Notch and Wnt basal components and cell cycle regulation	Li et al. (2007)
Melatonin	rescues early embryonic development by activating SIRT1/PGC-1α pathway	Niu et al. (2020)
*Per2* gene defects	breast branch morphogenesis	McQueen et al. (2018)

A study by Gotter et al. demonstrated that a possible mouse homology gene, *mimless* (*mtim*), is essential for embryonic development, and homozygous *mtim (−/−)* embryos die early in the uterus and developmental abnormalities are found as early as E5.5 embryos, suggesting that *mtim* is important before and after embryonic implantation (Gotter et al., 2000; Xiao et al., 2003). A study by Hanbin Dan et al. established the role of the fetal circadian clock as a regulator of the developmental timer, the number of renal units, and the branching rate, thus playing a fundamental role in kidney development (Dan et al., 2020). The circadian rhythm gene *Timeless* is involved in epithelial morphogenesis during kidney development (Li et al., 2007). Studies have reported that normal kidney development requires a complete fetal clock. Structural abnormalities found in *Bmal1* tissue-specific mutations and overall mutations in the kidney suggest that circadian clock dysfunction may contribute to congenital defects. Disruption of the fetal renal rhythmic expression of genes is also related to mammalian phenotypes (Dan et al., 2020) Local or integral deletion of fetal *Bmal1* produces recognizable phenotypic abnormalities and disrupts clock-correlated oscillatory expression of key developmental genes. The fetal clock described herein can provide a layer of regulation that coordinates the time of a variety of developmental processes (Dan et al., 2020). High-resolution 3D analysis of embryonic kidneys indicates that fetal clock interruptions, whether local or global, can lead to phenotypic defects, similar to congenital urinary tract abnormalities and congenital kidneys, which account for more than 50% of kidney failures and are a group of malformations that lead to child transplantation and dialysis (Dugoff, 2002; Verbitsky et al., 2019). These studies indicate that circadian clock transcription factors play an important role in the regulation of kidney branching morphogenesis, growth, and development (Dan et al., 2020).

Melatonin is secreted primarily by the pineal glands and is a multifunctional hormone regulated by circadian rhythms. It also plays a key role in embryonic development, and oocyte fertilization and maturation (Yong et al., 2021). Photoperiod information is converted from the pineal gland to rhythmic secretion of melatonin, which is released into the circulatory system at high concentrations at night and at low concentrations during the day. The rhythm of melatonin is controlled by the circadian clock and is considered an important output of the circadian rhythm system (Sánchez-Vázquez et al., 2019). Melatonin, a neuroendocrine hormone secreted by the pineal gland, transmits information about the ambient photoperiod (seasons and circadian rhythms) to relevant organs and tissues in the body (Hunt and Sassone-Corsi, 2007). Studies have reported that melatonin can protect against the development of vitrified embryos, which is related to its increased endogenous glutathione levels, anti-apoptosis, and direct free radical scavenging activity (Gao et al., 2012). Recent studies have also shown that melatonin affects gonadal development in mice (Kasahara et al., 2010), as well as the formation of teeth, bones, adrenal glands, and testicles (Torres-Farfan et al., 2006; Kasahara et al., 2010). Melatonin secreted by the salivary glands of embryos can affect organ development. In the early embryonic stage, melatonin maintains the shape of the salivary glands until the beginning of the branch morphology. In addition, the circadian rhythm of melatonin may affect the development of salivary glands (Obana-Koshino et al., 2015). Melatonin supplementation has been shown to significantly improve mitochondrial dysfunction, DNA damage, and zearalenone-induced oxidative stress early in embryonic development (Xu et al., 2019). Niu et al. showed that the beneficial effects of melatonin on early embryos depend on two crucial aspects. First, melatonin can prevent excessive mitochondrial biosynthesis induced by hyperoxia environments under sub-physiological conditions (Niu et al., 2020). Second, melatonin therapy protects fetal growth, placental perfusion, and fetal cardiovascular function during pregnancy (Itani et al., 2016). In malnourished pregnant rats, maternal melatonin supplementation increased birth weight (Richter et al., 2009). Therefore, the regulation of circadian rhythm disturbances may play an important role in the normal development of embryos.

## Effects of the circadian clock on biological processes after birth

**FIGURE 2:**
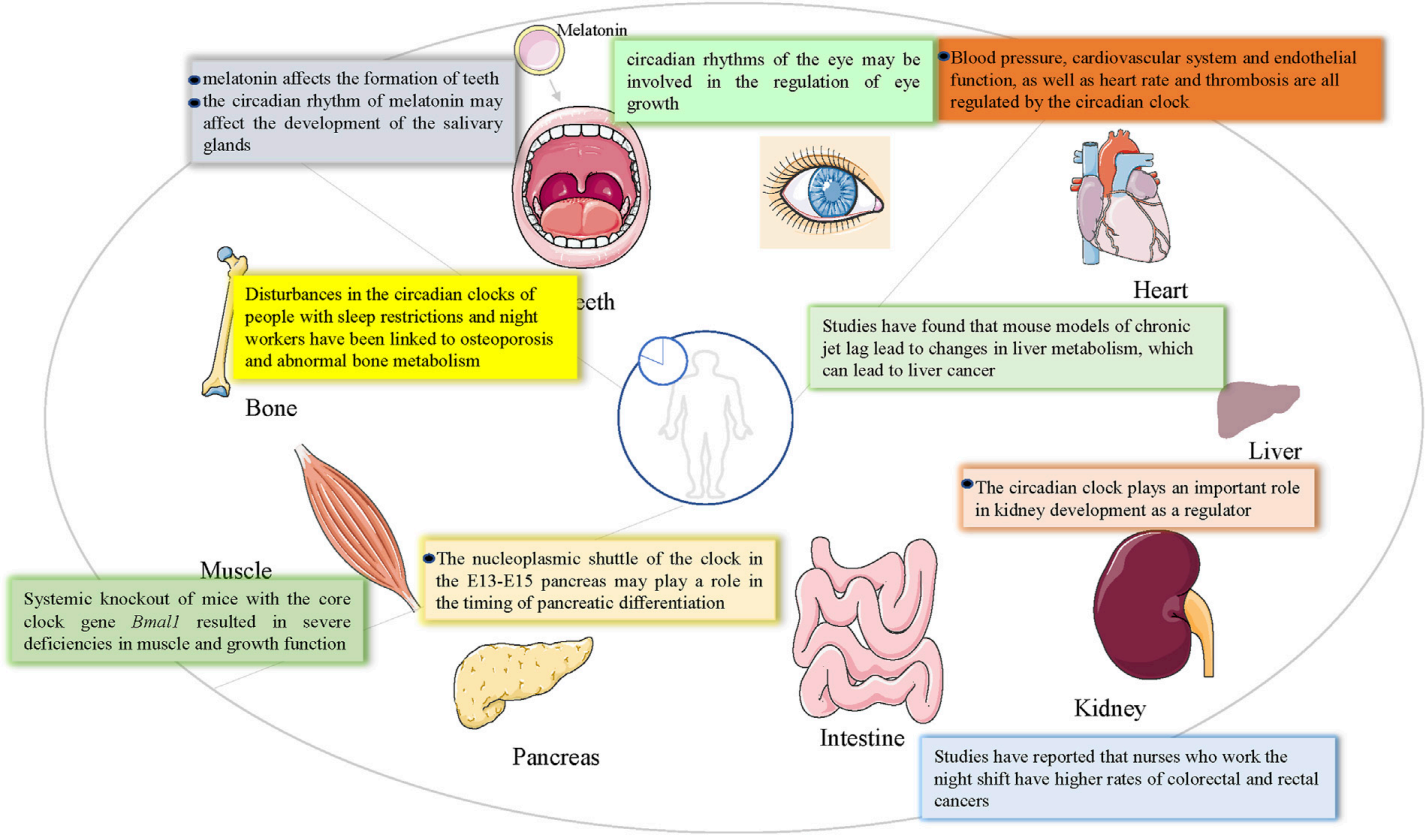
The influence of the circadian clock on various organs of the body.

Jennette P. Moreno et al. argued that children exhibit healthy seasonal patterns of height and weight gain controlled by an annual clock, whereas the summer vacation environment may favor circadian rhythm disorders caused by changes in social needs, promoting accelerated weight gain in children during the summer months (Moreno et al., 2019). There is evidence that weight gain and growth (height) in children are seasonal, suggesting that an endogenous rhythm of weight gain and growth exists in children (Bogin, 1978; Bogin, 1979). Since *Bmal1* and *Clock* downregulate many genes other than *Crys* and *Pers* (10% of transcription genes), daily rhythms, such as sleep, are created (Buhr and Takahashi, 2013). Individuals such as shift workers experience circadian rhythm disorders frequently and/or chronically because their waking/sleeping and eating/fasting cycles are out of sync with the circadian timing system (Morris et al., 2012). When social needs such as school or work require individuals to live according to a schedule that does not conform to their inner rhythm, it can make it difficult to wake up and fall asleep during socially mandated work or school hours. Social jet lag is an example of a chronic circadian rhythm disorder. This leads to unmet sleep needs, which can accumulate sleep debt (for example, social jet lag) during work or school. On weekends, different waking times may bring more variability in sleep, meals, and activity patterns, resulting in a disorder of peripheral and body central clocks, leading to negative health consequences, such as cardiovascular disease, obesity, and cancer (Garaulet et al., 2005; Laermans and Depoortere, 2016; Roenneberg and Merrow, 2016). Disorders of circadian rhythms can lead to serious health problems such as metabolic syndrome, organ fibrosis, aging, cancer, and osteopenia (Egstrand et al., 2020) (Figure 2). Studies have shown that normal rhythmic damage favors gene expression and activation of pro-inflammatory responses, which may also play a role in neurodegenerative changes (De Lazzari et al., 2018). It has also been shown that 2%–10% of mammalian genes are clock-controlled, and that they are mostly related to organ function and exhibit tissue-specific expression (Chu et al., 2011). Studies have shown that the circadian clock function is important for the cell cycle, *in vivo* tumor suppression, and DNA damage responses (Fu and Lee, 2003). Several studies have reported the expression of circadian clock genes in reproductive tissues, including the ovaries (Sellix and Menaker, 2010). Female *Clock*-mutant mice have irregular estrous cycles and carry 51 amino acid deletions in the transcriptional activation region of the clock gene, without a normal luteinizing hormone surge in the pre-estrus period (Miller et al., 2004). Blood pressure, the cardiovascular system, endothelial function, heart rate, and thrombosis are all regulated by the circadian clock (Hu et al., 2004). Twenty-four-hour rhythm disturbances can lead to cardiovascular diseases, including fibrosis, heart failure, arrhythmias, and myocardial infarction (Martino et al., 2008). In Dahl salt-sensitive rats fed a high-salt diet for 6 weeks, the development of hypertrophy was associated with decreased diurnal expression of the core clock gene of the heart (Mohri et al., 2003). Similarly, hamster models with ubiquitous circadian cycle changes can develop accelerated senile cardiac kidney disease (Martino et al., 2008). Consistent with these rodent models of circadian dysregulation, shift workers are at a significantly higher risk of cardiovascular disease (as well as various features of cardiometabolic syndrome) (Knutsson, 1995; Härmä and Ilmarinen, 1999). Circadian clock genes play a key role in dementia and neurodegenerative diseases (Cronin et al., 2017; Vaccaro et al., 2017). Studies have shown that long-term levodopa therapy may impair circadian rhythm function and lead to cognitive dysfunction. In this regard, simulating long-space flights alters circadian rhythms, leading to potential neuronal damage and cognitive decline (Wu et al., 2017). Circadian rhythm disorders that lead to sleep fragmentation may lead to the further progression of neurodegenerative diseases. Circadian disruption and sleep disorders exacerbate neurodegeneration, which in turn can disrupt circadian rhythms and sleep (Shen et al., 2023). Increasing data suggest circadian rhythm plays a crucial role in regulating metabolism and neurological diseases and Bmal1 is considered to be a key regulator of circadian transcription (Gao et al., 2023). Compared with C57BL/6 J mice, BMAL1 KO showed a significantly reduced seizure threshold (Gerstner et al., 2014). Deletion of Per2 gene disrupts the circadian rhythm system, resulting in dysregulation of the HPA stress axis, which is subsequently associated with increased depression-like behavior and deficient starling response (Russell et al., 2021). Circadian rhythm alterations are commonly reported in individuals with psychiatric disorders, including major depressive disorder (MDD), bipolar disorder (BD), anxiety, and schizophrenia (SZ). In turn, circadian disruption may trigger or exacerbate symptoms in individuals with a predisposition for mental health disorders (Walker et al., 2020). Cerebrospinal fluid promotes the entire brain through a lymphatic pathway involving the perivascular network, which is necessary to clear metabolic waste products during sleep. This system relies on glial cells, and sleep deprivation can lead to the development of neurodegenerative diseases and dysfunction of the system (Benveniste et al., 2017). Alterations in circadian clock genes have been shown to cause age-related cognitive and motor impairments, as well as the progression and onset of Alzheimer’s disease (AD) (Maiese, 2021). A large number of studies have shown that aging is accompanied by the weakening of circadian rhythm and the change of sleep pattern (Stowe and McClung, 2023). For example, sleep-wake disruptions may be associated with an increase in Alzheimer’s disease-related biomarkers. AD is characterized by dampened circadian rhythms with a delay in circadian phase and overall decrease in amplitude (Stowe and McClung, 2023). Aging is associated with dysregulation of the circadian system and cognitive decline (Adler et al., 2019). Circadian disruption has been linked to hippocampal dependent memory impairment, and it is conceivable that age-induced changes in circadian regulation, a key process in hippocampal function, could contribute to this decline. The current study further supports the key role of circadian molecules in regulating cognitive processing and ROS/RNS homeostasis. Loss of normal sleep/wake cycles is a leading cause of hospitalization for dementia and has even been considered a contributing factor and/or preclinical symptom of neurodegenerative diseases such as Alzheimer’s and Huntington’s disease (Gerstner, 2010).

Different tissues have different clock-rhythmic output genes, allowing circadian rhythm control of the proper activity and function of each organ (Casey et al., 2021). In breast tissue, the time expression pattern of the clock gene of the core protein changes with changes in the female reproductive state, which seems to be related to changes in the stage of gland development (Casey et al., 2014). The circadian clock relies on the modulation of circadian clock genes, and mice with *Per2* defects exhibit fewer bifurcations, and lack of catheter-deficient distal migration is evidence of the involvement of *Per2* in breast branch morphogenesis (McQueen et al., 2018) (Table 2). *Clock* mutant mice exhibit lactation defects (Hoshino et al., 2006). In addition, the reduction in postnatal survival in *Clock-△19* mice is associated with impaired breast development (Casey et al., 2021). In epidermal stem cells, it has been demonstrated that the circadian clock can adjust the Wnt/β-catenin pathway (Janich et al., 2011). Alterations in gene expression levels in the Wnt pathway may be a potential cause of the impaired mammary epithelial stem cell function observed in *Clock-△19* mice (Yang et al., 2017) and significant premature aging in *Bmal1−/−* mice (Gimble et al., 2009). *Bmal1* is necessary for bone marrow stem cell survival and growth (Burgermeister et al., 2019). Systemic knockout of mice with the core clock gene *Bmal1* results in severe deficiencies in muscle and growth function (Kondratov et al., 2006; Andrews et al., 2010). Studies have shown that CAMK-1 deletion strains from ascomycete germination grow slowly, whereas analysis of CAMK-1 deletion strains has shown that CAMK-1 deletion affects the cycle of conidia circadian rhythm, phase, and light that induce phase shifts (Yang et al., 2001). Gene deletions of core circadian transcription factors (TFs), *Clock* and *Bmal1,* lead to a significant shortening of lifespan and premature aging (Maryanovich and Frenette, 2016). Research by Ebert et al. and others showed that the core circadian factor *Bmal1* and *Clock* are necessary for the self-renewal and growth of leukemia stem cells (Puram et al., 2016). Data from Dapergola et al. suggested that disruption of molecular clocks or altered light conditions leads to alterations in INR/TOR signals that have an impact on the growth and proliferative properties of neural stem cells in the brains of developing *Drosophila* (Dapergola et al., 2021). In addition, mice with *Per2* mutations are susceptible to cancer, whereas ectopic expression of *Per1* and *Per2* can lead to growth inhibition (Gery et al., 2005; Gery et al., 2006). The PER protein binds to nuclear receptors, such as REV-ERBα and PPARα, regulating life activities, including growth and development, through nuclear receptor function (Grimaldi et al., 2010; Schmutz et al., 2010). In mice treated with 6-OHDA, mutations in the circadian clock element mEV-ERBα (*mBmal1* transcriptional inhibitory factor) promoted 6-OHDA-dependent motor deficits, neuroinflammation in the spinal midbrain, and dopaminergic neurodegeneration (Kim et al., 2018). Aged *Per* ^*0*^ mutant flies exhibited impaired motor ability, increased sensitivity to oxidative damage, and expanded vacuolarization in the central brain. The loss of functional *dPer* exacerbates damage from neurodegenerative predisposition mutants, suggesting that in *Drosophila*, the Clock gene acts as a protective factor during aging (Krishnan et al., 2009). Reproductive dysfunction has been studied in *Bmal1* ^*−/−*^ mice and mice with *Clock* mutations (Chu et al., 2011). Studies showed that mouse *Per2* or *Per1* loss-of-function mutations have a lower reproductive success rate in 9-month-old male mice (Pilorz and Steinlechner, 2008). Y ating Zheng et al. found that the number of follicles in *Per2/Per1* mutants decreased significantly from mid-year onward, compared to that is the litter control group. *Per2/Per1* deletion mutations may alter the expression of genes involved in follicle development, leading to a decreased reproductive capacity and depletion of follicular reserves (Zheng et al., 2019). Studies have shown that deletion of *Per2* or *Per1* affects fertilization rates in females aged 9–12 months, manifested by decreased implantation success rates and abnormal estrous cycles (Pilorz and Steinlechner, 2008). *Clock-△19* mice exhibited a point mutation that led to downregulation of the *Clock-Bmal1* target gene, which was manifested by a decrease in lactation ability, with an increase in neonatal mortality and a decrease in growth rate in mice (Dolatshad et al., 2006). Using shRNA to reduce clock proteins in breast epithelial cell lines affected cell growth and reduced the expression of factors related to milk synthesis and breast differentiation (Casey et al., 2021). *Bmal1* gene-deficient mice have a severely shortened lifespan and are sterile, weighing less than wild-type mice, and exhibit various characteristics of premature aging, such as arthropathy and early cataracts (Lee et al., 2010). Accelerated aging is a prominent feature in animal models with modified and missing core clock genes (Uth and Sleigh, 2014). Studies have reported that a non-coding polymorphism in the 3-UTR region of the human clock gene is linked to adult hyperactivity disorder and attention deficit (Kissling et al., 2008).

**TABLE 2:** Effects of the circadian clock on biological processes after birth.

Mouse/Experiment/Mutation	Phenotype/mechanism	References
increased expression of the circadian rhythm gene *hClock*	promote tumorigenesis (such as metastasis of colorectal cancer)	Wang et al. (2017)
*Per2* mutations	are susceptible to cancer	Gery et al. (2005)
*Per2* or *Per1* mutations	have a lower reproductive success rate in 9- month-old male mutants	Pilorz and Steinlechner (2008)
*Per2/Per1* deletion mutations	may alter the expression of genes involved in follicle development	Zheng et al. (2019)
*Bmal1* gene-deficient mice	have a severely shortened lifespan and are sterile, weighing less than wild-type mice	Lee et al. (2010)
The 5-repeat variant allele of the human *Per3* gene 4/5 repeat polymorphism	a tripled risk of breast cancer in premenopausal women	Uth and Sleigh (2014)
*Clock* mutant mice	exhibited significant structural muscular pathology	Miller et al. (2007)
Ablation of *Bmal1* or *Clock*	can lead to altered sarcosmosamus fibrous tissue and disruption of muscle contractility	Andrews et al. (2010)
Col2a1-Bmal1^−/−^	Age-related progressive knee cartilage degeneration and extracellular matrix loss	Dudek et al. (2016)
*Per2*, *Per1* as well as ROR deficiency	associated pathologies such as muscle movement and contraction defects	Zheng et al. (1999)
Bmal1^−/−^	decreased expression of Alp, Ihh and Col10a1 in rib growth plate	Takarada et al. (2012)
ClockΔ19	progressive proteoglycan loss in knee cartilage	Yuan et al. (2019)
Bmal1^osc−/−^	high bone mass and reduced bone resorption	Xu et al. (2019)
Bmal1^osx−/−^	low bone mass and increased bone resorption	Takarada et al. (2012)
Per1^−/−^Per2^−/−^	high bone mass	Fu et al. (2005)
Per1^−/−^Per2^m/m^	increased number of osteoblasts, mineral apposition and bone formation rate	Fu et al. (2005)
Per2^m/m^	high bone mass and increased osteoblast number and bone formation rate	Maronde et al. (2010)
Cry2^−/−^	high bone mass and decreased osteoclast activity	Maronde et al. (2010)
Rev-erb alpha and Rev-erb beta knockout	Impaired muscle maintenance and myogenic differentiation	Chatterjee and Ma (2016)
*Per1* knockout	increased the expression of MMP9 and MMP2, reduced the expression of TIMP-2, which may lead to the observed increase in tumor cell invasion and migration	Li et al. (2016)
Bmal1	controls the transcriptional activity of HIF1α and, once removed, causes nucleus medullary growth to be inhibited	Suyama et al. (2016)
Bmal1	plays a key role in cartilage development by modulating the Hif1α-vascular endothelial cell growth factor signaling pathway	Ma et al. (2019)
conditioned loss of *Bmal1*	resulted in shorter tibia and femur lengths than control mice, with growth retardation, which was associated with reduced expression of Indian hedgehog (IHH)	Takarada et al. (2012)
Bmal1	binds directly to the Opg promoter and upregulates its expression to inhibit the differentiation of osteoclasts	Zhou et al. (2018)

The circadian clock signal plays a key role in adjusting the mineralization and thickness of tooth enamel (Said et al., 2020). Immunohistochemical (IHC) studies have shown that *Clock*, *Per1*, *Bmal1*, and *Per2* proteins are expressed in developing dental organs (Zheng et al., 2011). The circadian clock regulates a wide range of physiological processes (King and Takahashi, 2000; Schibler and Sassone-Corsi, 2002) including the processes by which other mineralized tissues affect bone growth and homeostasis (Fu et al., 2005). The periodicity observed in enamel suggests that the molecular basis that coordinates enamel development may be affected by the circadian clock (Lacruz et al., 2012). To determine the clock gene and diurnal rhythm of Amelx, Rodrigo et al. used mouse glazed cells for serum synchronization and analyzed the expression of circadian transcription factors *Bmal1* and *Per2* using real-time quantitative polymerase chain reaction (qPCR). The results showed that these crucial gene regulators of the circadian clock were expressed in synchronized cultured mouse glazed cells, and their expression patterns followed the reverse circadian rhythm pattern of the two gene transcripts (Lacruz et al., 2012). The ablation of the superopedial nucleus in rats led to the destruction of the incremental line in dentin (Anresen line), supporting the participation of the circadian clock in the formation of this tissue (Ohtsuka-Isoya et al., 2001). Rodrigo et al. found that in cultured glazed cell LS8, the clock genes *Bmal1* and *Per2* oscillate in reverse over a 48-h period. They also demonstrated the expression of *Cry1* and *Bmal1* in developing glazed cells (Lacruz et al., 2012). In addition, they measured daily fluctuations in the expression of amelogenin (Amelx) in the entire developing mouse tooth grinding process, which is a specific product of amelo-blast cell differentiation (Lacruz et al., 2012). Immunocytochemical (ICC) results showed that the expression of amelx and melatonin receptors (MTS) was markedly delayed in newborn mice grown in a full-light or all-dark environment, as well as during enamel development. The development of tooth enamel was also delayed, showing significant immature histology (Tao et al., 2016; Li et al., 2007). Studies have shown that the disruption of SOCE (store operated Ca2+ entry) significantly affects the molecular circadian clock of glazed cells, suggesting that alterations in circadian clocks may be associated with the development of stromal interaction molecule 1 (STM1)-mediated AI (amelogenesis imperfecta) (Said et al., 2020).

**FIGURE 3:**
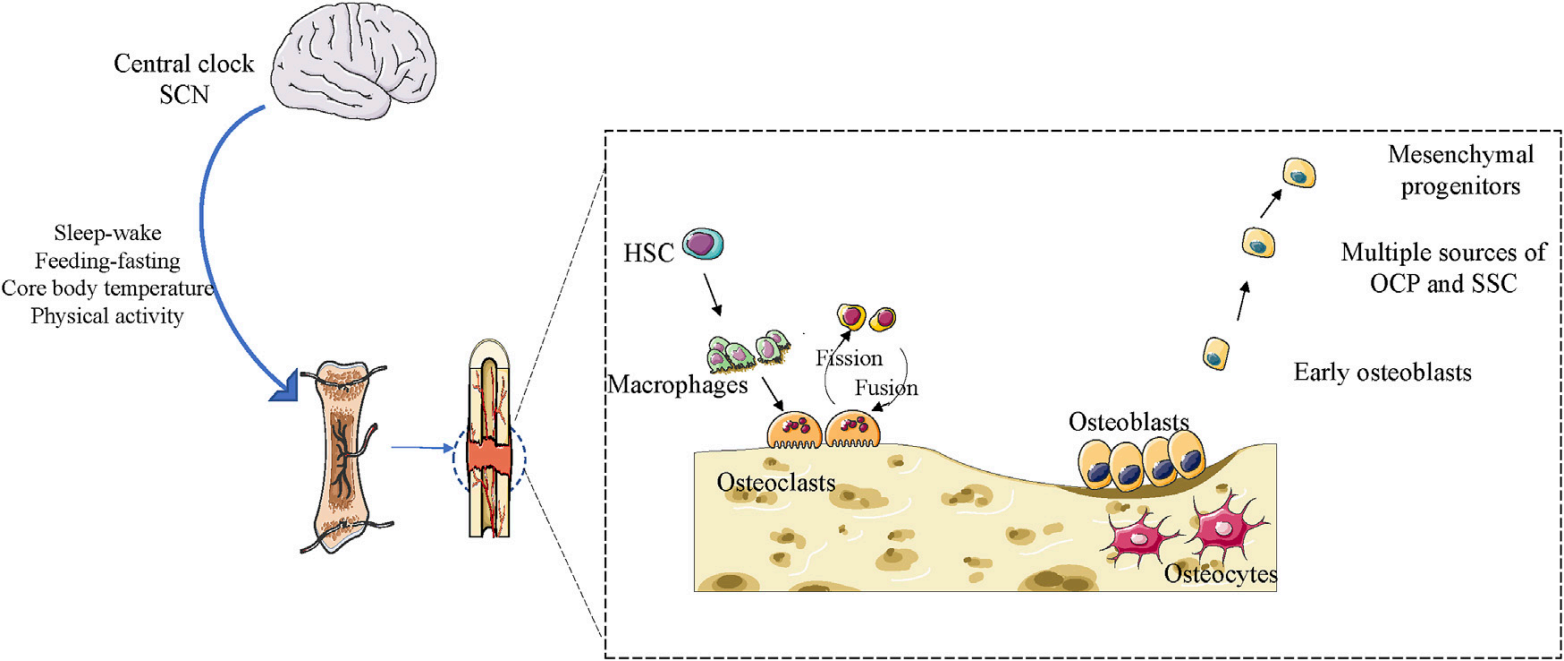
Effect of the circadian clock on bone remodeling in adulthood in maintaining bone integrity and homeostasis.

Bone is a dynamic tissue that constantly responds to environmental changes such as mechanical stress and nutrition (Chan et al., 2021). Bone homeostasis in adult life is maintained through bone reconstruction, which is a balanced and controlled process involving bone resorption by osteoblasts and osteoclasts (Chan et al., 2021). Over the past decade, several studies have revealed complex interactions between skeletal systems and the circadian clock (Gonçalves and Meng, 2019). Disturbances in the circadian clock of people with sleep restrictions and night workers have been linked to osteoporosis and abnormal bone metabolism (Feskanich et al., 2009). Osteoblasts at the site of fracture healing and tissue homeostasis are affected by the circadian clock (Chan et al., 2021) (Figure 3). The timing of eating and circadian rhythm disorders can alter bone metabolism (Egstrand et al., 2020). In recent years, from structural maintenance to functional regulation, several studies have reported that the clock system in skeletal muscle plays a key role in critical aspects of skeletal muscle physiology (Andrews et al., 2010; Chatterjee et al., 2013; Chatterjee et al., 2015). Studies in mice with mutations in the clock gene have shown a clear link between maintaining the entire molecular clock circuitry and skeletal muscle growth and development (Chatterjee and Ma, 2016). In vitro culture studies of flat and long bones, *Per2* was found to have a 24-h rhythmic expression at the site of fracture healing (Kunimoto et al., 2016). Studies have reported that *Bmal1* deficiency can lead to severe aging-related osteoporosis, and at 9 months of age, the loss of the *Bmal1* gene leads to an approximate 50% weight loss (Kondratov et al., 2006). *Clock* mutant mice exhibited significant structural muscular pathology, skeletal muscle weakness, altered muscle filament structure, and altered mitochondrial volume (Miller et al., 2007). Molecular clocks play an important role in maintaining correct time regulation of muscle clock control genes (CCGs). In the first stage of myoblast differentiation and growth, myogenic differentiation 1 (MyoD1) is activated and becomes CCG dependent on its unique diurnal expression trends in adult muscles (Riley and Esser, 2017). Ablation of *Bmal1* or *Clock*, key elements of the molecular clock, impairs its target genes and circadian expression of MyoD1, which can lead to altered sarcosmosamus fibrous tissue and disruption of muscle contractility (Andrews et al., 2010). The modulation of myogenic progenitor cells by *Bmal1* has been shown to be imperative for the regeneration of muscle tissue after injury. In the muscle injury-induced regeneration model, animals lacking *Bmal1* exhibited defects in the regenerative muscle generation process, accompanied by alterations in the process of satellite cell expansion and a decrease in repair capacity (Chatterjee et al., 2015). In mice with *Clock* mutations and *Bmal1* gene defects, the finding of similar functional defects produced by muscle-specific forces suggests that clocks play a synergistic role in this basic skeletal muscle function (Bunger et al., 2000; Kondratov et al., 2006). In addition, mice with ROR, *Per1*, and *Per2* deficiency, were found to have associated pathologies such as muscle movement, contraction defects, and weakness in muscle function and structure (Zheng et al., 1999; Zhang et al., 2015). Studies have shown that adult *Bmal1* gene deficient mice may experience significant muscle weight loss due to impaired hypertrophic growth and developmental defects (Chatterjee and Ma, 2016). The opposite effects of the clock suppressor *Rev-erbα* and activator *Bmal1* on myogenic development and the effect of RoRα on myogenic pathways strongly suggest the role of circadian clock genes in regulating myogenic precursor development (Chatterjee and Ma, 2016). Jeffrey et al. measured muscle growth in live zebrafish larvae over a 12-h period and found that muscles grew more during the day than at night. The expression of dominant negative CLOCK (△clk) reduces muscle growth, inhibits molecular clock function, and eliminates circadian rhythm differences (Kelu et al., 2020). Another important insight provided by growth plate research is the influence of the circadian clock on developmental signaling pathways and its regulation of osteogenesis in cartilage (Samsa et al., 2017). Some chondrocyte-specific genes, such as Col10a1 and Col2a1, are expressed in a diurnal pattern in ATDC5 chondrocytes, as well as in growth plates and articular cartilage, in addition to core clock genes (Gossan et al., 2013; Okubo et al., 2013). Parathyroid hormone (PTH) can reset the body clock of the femur in a dose- and time-dependent manner (Hanyu et al., 2011; Okubo et al., 2015). PTH modulates the expression of mouse *Per2* and *Per1* through the PKA-CREB signaling pathway (Hinoi et al., 2006). Therefore, there may be a peripheral clock specific to chondrocytes that is essential for growth plate development. In mice with *Bmal1* gene defects, Ihh-mediated chondrocyte differentiation is impaired, and in *Bmal1* gene-defective mice, the tibia and femur are significantly shortened (Takarada et al., 2012; Samsa et al., 2016). In addition, chondrocyte-specific removal of *Bmal1* led to severe cartilage degeneration in 3-month-old rats (Samsa et al., 2017). One study showed that conditional knockout of *Bmal1* in cartilage affects the integrity of cartilage and makes joints more susceptible to osteoarthritis (Dudek et al., 2016). Another study indicated that in mouse nucleus medullary cells, *Bmal1* controls the transcriptional activity of HIF1α and, once removed, inhibits nuclear medullary growth (Suyama et al., 2016). Zheng et al. found that bmal1 plays a key role in cartilage development by modulating the Hif1α-vascular endothelial cell growth factor signaling pathway (Ma et al., 2019). Ma et al. demonstrated that the deletion of a specific *Bmal1* in postnatal cartilage leads to decreased chondrocyte proliferation, disorders of cartilage formation in growth plates, and hypertrophy of chondrocytes that hinder the growth of longitudinal bone (Ma et al., 2019). Another study showed that a conditioned loss of *Bmal1* in chondrocytes resulted in shorter tibia and femur lengths than control mice, with growth retardation associated with reduced expression of Indian hedgehog (IHH) (Takarada et al., 2012). Studies have identified the core circadian rhythm gene *Bmal1* as being essential for maintaining normal intrachondral osteogenesis (Ma et al., 2019), and have shown that *Bmal1* binds directly to the OPG promoter and upregulates its expression to inhibit osteoclast differentiation (Zhou et al., 2018).

There is growing evidence that failure of the circadian clock system is correlated with the pathogenesis of cancer (Greene, 2012; Sahar and Sassone-Corsi, 2012). There are several examples illustrating the link between circadian rhythms, circadian rhythm genes, age-related phenotypes, and cancer (Lee et al., 2010). Studies have shown that mice with *Clock* mutations resulting in rhythmic loss exhibit many phenotypic abnormalities, such as an increase in the incidence of underlying cancer or an increase in the incidence of cancer after genetic stress (Lee et al., 2010). The results of Puram et al. revealed that the survival and growth of leukemia cells depend on clock mechanisms (Puram et al., 2016). Nurses who work night shifts have higher rates of colorectal, breast, endometrial, and rectal cancer (Schernhammer et al., 2001). This risk increases as shift hours increase (Papantoniou et al., 2016). An increased risk of prostate cancer was also observed in male night shift workers, and the aggressiveness of the disease was found to be associated with night shift time (Papantoniou et al., 2015). Studies have reported that people who travel by air have a higher risk of developing cancer, which is associated with jet lag. One meta-analysis showed that female flight attendants who often experience endogenous circadian rhythm disturbances have an increased risk of malignant melanoma and breast cancer (Tokumaru et al., 2006). Mouse models of chronic jet lag exhibit changes in liver metabolism, which can lead to liver cancer (Kettner et al., 2016). Studies have shown that an increasing number of diagnosed cases of hepatocellular carcinoma are thought to be related to circadian clock disorders, obesity and non-alcoholic fatty liver disease (Crespo et al., 2021). The occurrence and progression of liver cancer relies on multiple molecular mechanisms, involving genetic and epigenetic alterations of oncogenes and tumor suppressor genes, as well as disorder control, abnormal functions and inappropriate interactions of key molecular cascades such as Hedgehog, MAPK and PI3K/AKT/mTOR signaling pathways (Skrlec and Talapko, 2022). Studies have reported that decreased levels of melatonin or decreased excretion of its main metabolite, 6-thiooxidative melatonin, are associated with an increased risk of cancer, suggesting the anticancer effects of this indoleamine. Overall, published data strongly support the inhibitory effect of melatonin on different types of tumors, including liver cancer (Fernández-Palanca et al., 2021). The inhibitory effect of melatonin in liver tumors involves a variety of different cellular processes and molecules, including cell cycle arrest, decreased cell growth, limiting vascular metastasis and production, and promoting cell death. In addition, melatonin has been reported to increase the sensitivity of liver cancer cells to existing treatments (Prieto-Domínguez et al., 2017). In addition, increased expression of the circadian rhythm gene *hClock* may promote tumorigenesis by enhancing the expression of genes associated with angiogenesis, such as those involved in metastasis of colorectal cancer (Wang et al., 2017). The five-repeat variant allele of the human *Per3* gene 4/5 repeat polymorphism is associated with a triple risk of breast cancer in premenopausal women (Uth and Sleigh, 2014). Studies have reported that in breast cancer, the expression level of *Znf704* is inversely associated with the expression level of *Per2*, and high levels of *Znf704* are correlated with lymph node positivity, poor prognosis, and histological grading in patients with breast cancer (Yang et al., 2020). Baharan et al. revealed that *Bmal1* and P2-HNF4α are incompatible in liver cancer and that forcing the expression of *Bmal1* in HNF4α-positive liver cancer can stop tumor growth *in vivo* (Fekry et al., 2018). Studies have reported that PFKFB3 mediates circadian rhythm control of tumor growth, underscoring the significance of time-based PFKFB3 inhibition in cancer treatment (Chen et al., 2016). Studies have reported a lack of tumor suppression and cell cycle disorders in mice with *Per2* gene defects, suggesting that *Per2* plays a tumor-suppressive role through the DNA damage response pathway (Morgan et al., 2019). Another study found that *Per1* knockout increased the expression of MMP9 and MMP2 and also reduced the expression of TIMP-2, which may lead to the observed increase in tumor cell invasion and migration (Li et al., 2016). The immune system provides defense mechanisms to eliminate or curb the appearance of tumor cells in healthy tissues and prevent the further development of life-threatening cancers (Gajewski et al., 2013; Chen and Mellman, 2017). Both innate and adaptive immune responses exhibit circadian rhythms (about 24 h) (Scheiermann et al., 2018; Palomino-Segura and Hidalgo, 2021), There is evidence that the circadian clock components of cancer cells are disturbed, which drives cancer development (Papagiannakopoulos et al., 2016). Using immunodeficient mice and mice lacking lineage-specific circadian functions, study has found that CD8^+^ T cells and dendritic cells (DCs) exert circadian anti-tumour functions that control melanoma volume (Wang et al., 2023). Melanoma and Breast cancer mouse models show that circadian rhythm disturbance enhances cancer cell proliferation, stemness, spread and metastasis, induces immunosuppressive TME by increasing the proportion of TAM and regulatory T cells (TREGs), and promotes the differentiation of macrophages into anti-inflammatory phenotype, resulting in reduced infiltration of active CD8^+^ T cells (Aiello et al., 2020; Hadadi et al., 2020). RORγ activation inhibits tumor growth and prolongs survival by enhancing Th17 cell differentiation and function and reducing Treg levels (Hu et al., 2016).

## Summary and outlook

Circadian rhythms control several pathological and physiological processes, including various human diseases and embryonic development in mammals (Jensen and Cao, 2013; Dunlap et al., 2007). Targeting the components of circadian clock has received much attention as a novel approach to treating chronic diseases such as metabolic syndrome, chronic inflammatory diseases and cancer (Roenneberg and Merrow, 2016; Rijo-Ferreira and Takahashi, 2019). In theory, there are two drug approaches that could target the circadian clock: either by directly regulating the core circadian rhythm gene, or by targeting its regulators. However, since BMAL1 and CLOCK are transcription factors, it is challenging to directly target these circadian core genes (Takahashi, 2017; Trott and Menet, 2018). Therefore, pharmacological drugs that target proteins responsible for phosphorylating or degrading the clock components that negatively regulate BMAL1 and CLOCK have been developed as antagonists or agonists to disrupt the circadian network. Advances have been made in the chronopharmacology of those agents that target cancer, immunity, metabolism, coagulation, the cardiovascular system, and inflammatory and rheumatologic processes (Hermida et al., 2019; Tamimi et al., 2022; Civelek et al., 2023; Leung et al., 2023). Supplement dosing and schedule recommendations with circadian rhythm are recommended to improve drug safety and efficacy. The study of circadian drug responses has become a continuum from cell cultures to organoids, animal models, and patients (Lévi et al., 2024). Meaningful improvements in drug tolerability and/or efficacy through appropriate timing of administration have been demonstrated for immunotherapy and chemotherapy for cancer, as well as pharmacological agents commonly used in cardiovascular, metabolic, inflammatory, and neurological diseases (Lévi et al., 2024). However, few compounds have entered clinic trials that can evaluate the true efficacy of patients, and the dependence of these therapeutic effects on clock regulation still needs further investigation (Zhou et al., 2022).
